# *Dendrobium catenatum Lindl.* Water Extracts Attenuate Atherosclerosis

**DOI:** 10.1155/2021/9951946

**Published:** 2021-08-24

**Authors:** Jichun Han, Jing Dong, Rui Zhang, Xiaofeng Zhang, Minghan Chen, Xiangcheng Fan, Maoru Li, Jiajing Li, Junyi Zhu, Jing Shang, Yunyun Yue

**Affiliations:** School of Traditional Chinese Pharmacy, China Pharmaceutical University, Nanjing, 211198 Jiangsu, China

## Abstract

**Objectives:**

*Dendrobium catenatum Lindl*. (DH) is a Chinese herbal medicine, which is often used to make tea to improve immunity in China. Rumor has it that DH has a protective effect against cardiovascular disease. However, it is not clear how DH can prevent cardiovascular disease, such as atherosclerosis (AS). Therefore, the purpose of this study is to study whether DH can prevent AS and the underlying mechanisms.

**Methods:**

Zebrafish larvae were fed with high-cholesterol diet (HCD) to establish a zebrafish AS model. Then, we used DH water extracts (DHWE) to pretreat AS zebrafish. The plaque formation was detected by HE, EVG, and oil red O staining. Neutrophil and macrophage counts were calculated to evaluate the inflammation level. Reactive oxygen species (ROS) activity, malondialdehyde (MDA) content, and superoxide dismutase (SOD) activity in zebrafish were measured to reflect oxidative stress. The cholesterol accumulation and the levels of lipid, triglyceride (TG), and total cholesterol (TC) were measured to reflect lipid metabolism disorder. Then, parallel flow chamber was utilized to establish a low shear stress- (LSS-) induced endothelial cell (EC) dysfunction model. EA.hy926 cells were exposed to LSS (3 dyn/cm^2^) for 30 min and treated with DHWE. The levels of ROS, SOD, MDA, glutathione (GSH), and glutathiol (GSSG) in EA.hy926 cells were analysed to determine oxidative stress. The release of nitric oxide (NO), endothelin-1 (ET-1), and epoprostenol (PGI_2_) in EA.hy926 cells was measured to reflect EC dysfunction. The mRNA expression of intercellular adhesion molecule-1 (ICAM-1) and vascular cell adhesion molecule-1 (VCAM-1) in EA.hy926 cells was detected to reflect EC dysfunction inflammation.

**Results:**

The results showed that DHWE significantly reduced cholesterol accumulation and macrophage infiltration in early AS. Finally, DHWE significantly alleviate the lipid metabolism disorder, oxidative stress, and inflammation to reduce the plaque formation of AS zebrafish larval model. Meanwhile, we also found that DHWE significantly improved LSS-induced EC dysfunction and oxidative stress *in vitro*.

**Conclusion:**

Our results indicate that DHWE could be used as a prevention method to prevent AS.

## 1. Introduction

Atherosclerosis (AS) is characterized by endothelial dysfunction, inflammation, progressive lipid deposition, and vessel stiffness with potential complications, such as myocardial infarction or stroke [1, 2]. Hypercholesterolemia is an important risk factor for the occurrence and development of AS. Hypercholesterolemia raises blood lipid levels, causing lipids to accumulate in blood vessels, forming early AS plaques [3]. Hypercholesterolemia can also cause oxidative stress and inflammation and promote AS [4]. Due to the improvement of living conditions, people's diet has undergone great changes, and there are more and more high-cholesterol diets (HCDs), which significantly increase the occurrence of AS.

Clinical studies have shown that AS develops preferentially near branches and bends exposed to complex blood flow that generates shear stress with low-magnitude and significant variation in direction (e.g., oscillations and low shear stress) [5]. Shear stress is involved in the development of AS [6, 7]. Shear stress, a force between blood flow and blood vessel endothelium for the unit area of the blood vessel wall, is mainly divided into low shear stress (LSS), high shear stress, laminar shear stress, and oscillation shear stress. LSS can induce endothelial cell (EC) dysfunction and oxidative stress, eventually leading to plaque formation [8, 9]. LSS also promotes AS by priming ECs for enhanced expression of inflammatory molecules (e.g., intercellular adhesion molecule-1 [ICAM-1] and vascular cell adhesion molecule-1 [VCAM-1]) [10].

Studies have shown that atherosclerotic coronary artery disease is the main cause of cardiovascular disease morbidity and mortality, and the incidence of AS is increasing year by year, which seriously endangers people's life and health [11, 12]. Although the harm of AS is serious, the drugs for treating AS are very scarce. Currently, the drugs used to treat AS are mainly lipid-lowering drugs. However, since the pathogenesis of AS is complex, long-term and high-dose applications of single drug therapies, such as simvastatin, which targets a single molecule, can produce some side effects like myopathy and liver damage. Therefore, it is necessary to search for an effective therapeutic method to prevent or attenuate AS.

The main drugs currently used to treat AS are lipid-lowering drugs (such as statins), antiplatelet drugs (such as aspirin), and antioxidant drugs (such as probucol and vitamin C) [13–16]. Although these drugs can reduce the occurrence of AS, they still cannot meet the treatment of AS. Difficulty in eliminating plaques is the acknowledged shortcoming of these drugs. Many studies have found that some natural extracts have good anti-AS effects, such as mulberry and salvia miltiorrhiza [17, 18]. Because of the homology of medicine and food, these natural products are considered to be safe. For example, mulberry, which belongs to food, has extremely high safety. Therefore, it is feasible to find some plant extracts to prevent or treat AS.

*Dendrobium catenatum Lindl.* is a traditional Chinese medicinal plant species with anti-inflammatory, antioxidant, and hypolipidaemic effects, and it is also edible. As one herbal medicine component, it was originally applied as a tonic to cure cataracts, throat inflammation, fever, and chronic superficial gastritis in China [19–21]. However, its protective effect against AS has not been reported.

This study is aimed at evaluating the effects of *Dendrobium catenatum Lindl.* water extracts (DHWE) on AS in a AS zebrafish model. This work also reveals the effects of DHWE on EC dysfunction in HUVECs. Our findings demonstrate that DHWE effectively alleviate early AS symptoms, lipid levels, oxidative stress, and inflammation in zebrafish with AS as well as improve LSS-induced EC dysfunction. The results of this study indicate that *Dendrobium catenatum Lindl.* could be an effective drug for the treatment of early AS.

## 2. Materials and Methods

### 2.1. Test Compounds, Chemicals, and Reagents

*Dendrobium catenatum Lindl.* was purchased from Anhui Yuanhe Chinese Medicine Development Co., Ltd. (Hefei, Anhui Province, China). MTT kit was purchased from Sigma-Aldrich (St. Louis, MO, United States). The other chemicals and reagents were of analytical grade.

### 2.2. Zebrafish

Studies were performed using wild-type or transgenic zebrafish lines Tg(*fli1a*:EGFP) (endothelial EGFP), Tg(*lyz*:DsRED2) (macrophages are specifically labelled with red fluorescent protein), and Tg(*mpx*:EGFP) zebrafish (neutrophils are specifically labelled with green fluorescent protein) that were purchased from China Zebrafish Resource Center. Zebrafish were housed at 28.5°C under a 14 : 10 h light/dark cycle and fed with live brine shrimp twice daily.

### 2.3. Preparation of DH Water Extracts (DHWE)

Stems of DH were powdered using a pulveriser. In all, 25 g of DH powder was Soxhlet extracted using 500 mL of distilled water; extraction was performed twice for 1 h. The solution was filtered with a 200 mesh filter cloth, and the two extracts were combined. The aqueous extract was concentrated to 450 mL using a rotary evaporator and finally freeze-dried into powder.

### 2.4. HPLC Analysis of DHWE

The HPLC fingerprint of DHWE was examined with the Hitachi HPLC System (L-2000 series, Tokyo, Japan). We injected 10 *μ*L of extract (10 ng/*μ*L) into a reverse-phase column (Cosmosil 5C18-AR-II, 5 *μ*m, 25 cm × 4.6 mm I.D.), and the UV detection wavelength was set at 206 nm. DHWE were analysed with HPLC.

### 2.5. Zebrafish AS Model

According to our research [22], we adopted a high cholesterol diet (HCD) to establish a zebrafish AS model. Five-day old zebrafish larvae were fed an HCD enriched with 8% cholesterol for 45 days. After feeding HCD for 45 days, plaques will form in the blood vessels of zebrafish, resulting in vascular stenosis.

### 2.6. Detection of the Effect of DHWE on Plaque Formation in AS Zebrafish

The five-day old wild-type AB-line zebrafish larvae were randomly divided into five groups: control group, AS group, 0.1 mg/L DHWE group, 1 mg/L DHWE group, and 10 mg/L DHWE group. In the control group, five-day old zebrafish larvae were fed normal basic feed for 45 days. In the AS group, five-day old zebrafish larvae were fed an HCD enriched with 8% cholesterol for 45 days. In the various concentrations of DHWE (0.1, 1, and 10 mg/L) treatment groups: five-day old zebrafish larvae were fed an HCD enriched with 8% cholesterol for 45 days and treated with various concentrations of DHWE (0.1, 1, and 10 mg/L). According to our research [22], after 45 days of feeding, the zebrafish were completely fixed with paraformaldehyde, and the plaques in the blood vessels of each group of zebrafish were detected by HE staining (see the Supplementary Materials 1 for specific steps), EVG staining (see the Supplementary Materials 2 for specific steps), and oil red O staining (see the Supplementary Materials 3 for specific steps).

### 2.7. Detection of the Effect of DHWE on Cholesterol Accumulation in AS Zebrafish

The five-day old Tg(*fli1a*:EGFP) (endothelial EGFP) zebrafish larvae were randomly divided into five groups: control group, AS group, 0.1 mg/L DHWE group, 1 mg/L DHWE group, and 10 mg/L DHWE group. In the control group, five-day old Tg(*fli1a*:EGFP) zebrafish larvae were fed only with 10 *μ*g/g of red fluorescent lipid (without 8% cholesterol) for 10 days. In the AS group, five-day old Tg(*fli1a*:EGFP) zebrafish larvae were fed an HCD enriched with 8% cholesterol and supplemented with 10 *μ*g/g of red fluorescent lipid for 10 days. In the various concentrations of DHWE (0.1, 1, and 10 mg/L) treatment groups: five-day old Tg(*fli1a*:EGFP) zebrafish larvae were fed an HCD enriched with 8% cholesterol and supplemented with 10 *μ*g/g of red fluorescent lipid for 10 days and treated with various concentrations of DHWE (0.1, 1, and 10 mg/L). According to our research [22], images of the caudal vasculature in live larvae show diffuse red fluorescence of circulating fluorescent lipids in both the control and HCD-fed larvae and bright fluorescent lipid deposits in the blood vessel wall only in HCD-fed larvae. Studies have found that these accumulated lipids are similar to the plaques of early AS [23].

### 2.8. Detection of the Effect of DHWE on Macrophage Infiltration in AS Zebrafish

Studies have found that Tg(*lyz*:DsRED2) zebrafish (macrophages are specifically labelled with red fluorescent protein) can be used to observe inflammatory responses [24]. The five-day old Tg(*lyz*:DsRED2) zebrafish larvae were randomly divided into five groups: control group, AS group, 0.1 mg/L DHWE group, 1 mg/L DHWE group, and 10 mg/L DHWE group. In the control group, five-day old Tg(*lyz*:DsRED2) zebrafish larvae were fed only normal basal feed (without 8% cholesterol) for 10 days. In the AS group, five-day old Tg(*lyz*:DsRED2) zebrafish larvae were fed an HCD enriched with 8% cholesterol for 10 days. In the various concentrations of DHWE (0.1, 1, and 10 mg/L) treatment groups: five-day old Tg(*lyz*:DsRED2) zebrafish larvae were fed an HCD enriched with 8% cholesterol for 10 days and treated with various concentrations of DHWE (0.1, 1, and 10 mg/L). Macrophages marked with green fluorescence in blood vessels can be directly observed under a fluorescence microscope.

### 2.9. Detection of the Effect of DHWE on Lipid Levels in AS Zebrafish

The five-day old wild-type AB-line zebrafish larvae were randomly divided into five groups: control group, AS group, 0.1 mg/L DHWE group, 1 mg/L DHWE group, and 10 mg/L DHWE group. In the control group, zebrafish larvae were fed only normal basal feed (without 8% cholesterol) for 10 days. In the AS group, zebrafish larvae were fed an HCD enriched with 8% cholesterol for 10 days. In the various concentrations of DHWE (0.1, 1, and 10 mg/L) treatment groups: zebrafish larvae were fed an HCD enriched with 8% cholesterol for 10 days and treated with various concentrations of DHWE (0.1, 1, and 10 mg/L). After 10 days of feeding and fasting for 24 h, Nile red staining was used to detect lipid levels in each group of zebrafish. The stock solution (1.25 mg/mL) of Nile red (Invitrogen N-1142) was prepared in acetone and stored in the dark at 20°C. For the staining of fish, the stock solution was diluted to 50 ng/mL in egg water and incubated for 15 min at 28°C in the dark. The fish were washed with distilled water 3 times and anaesthetized with a few drops of tricaine stock solution (Sigma; 4 mg/mL, pH 7.0). The fish were mounted in 4% methylcellulose, and Nile red staining was imaged under Olympus SZX16 (Olympus Corporation, Japan) Microscope, which was used for yellow fluorescent imaging.

### 2.10. Detection of the Effect of DHWE on ROS Activity in AS Zebrafish

The five-day old wild-type AB-line zebrafish larvae were randomly divided into five groups: control group, AS group, 0.1 mg/L DHWE group, 1 mg/L DHWE group, and 10 mg/L DHWE group. The administration of each group of zebrafish is consistent with that described in Section 2.9. After 10 days of feeding and fasting for 24 h, DCFH-DA was used to detect ROS expression in each group of zebrafish.

### 2.11. Biochemical Measurement

The five-day old wild-type AB-line zebrafish larvae were randomly divided into five groups: control group, AS group, 0.1 mg/L DHWE group, 1 mg/L DHWE group, and 10 mg/L DHWE group. The administration of each group of zebrafish is consistent with that described in Section 2.9. After 10 days of feeding and fasting for 24 h, 5 larvae from each group were randomly selected and sacrificed as one sample, and six samples were prepared for testing each index. Triglyceride (TG) levels, total cholesterol (TC) levels, superoxide dismutase (SOD) activity, and malondialdehyde (MDA) levels were measured by commercial assay kits (Jiancheng, Nanjing, China), following the manufacturer's instructions. All the quantitation of the above kits was read by a multifunctional microplate reader.

### 2.12. Detection of the Effect of DHWE on Inflammation in AS Zebrafish

Studies have found that Tg(*mpx*:EGFP) zebrafish (neutrophils are specifically labelled with green fluorescent protein) can be used to observe inflammatory responses [25], and we observed inflammation in Tg(*mpx*:EGFP) zebrafish. The five-day old Tg(*mpx*:EGFP) zebrafish larvae were randomly divided into five groups: control group, AS group, 0.1 mg/L DHWE group, 1 mg/L DHWE group, and 10 mg/L DHWE group. In the control group, zebrafish larvae were fed only normal basal feed (without 8% cholesterol) for 10 days. In the AS group, zebrafish larvae were fed an HCD enriched with 8% cholesterol for 10 days. In the various concentrations of DHWE (0.1, 1, and 10 mg/L) treatment groups: zebrafish larvae were fed an HCD enriched with 8% cholesterol for 10 days and treated with various concentrations of DHWE (0.1, 1, and 10 mg/L). After 10 days of feeding and fasting for 24 h, the number of neutrophils was observed under a fluorescence microscope to reflect inflammation in zebrafish.

### 2.13. Cell Culture

The human umbilical vein endothelial cell (HUVEC) line EA.hy926 cells (American Type Culture Collection) were cultured in DMEM (Invitrogen) containing 10% heat-inactivated foetal bovine serum (FBS), penicillin (100 U/mL), and streptomycin (100 *μ*g/mL) at 37°C in a 5% CO_2_ humid incubator. When the EA.hy926 cells grew to log phase, the cells were seeded onto a glass slide (30 × 50 mm) and treated with DMSO (0.1%) or various concentrations of DHWE (0.1, 1, and 10 mg/L) for 24 h. After 24 h, the LSS test was started.

### 2.14. Low Shear Stress

A parallel flow chamber (Shanghai Medical Instrument School, Shanghai, China), which consists of two stainless steel plates and a silicon gasket, was used in this study. A glass slide (30 × 50 mm) with confluent cells was placed on the lower plate of the chamber and then subjected to LSS induced by continuous fluid flow. Shear stress values (3 dyn/cm^2^) were modulated by the flow through the chamber.

### 2.15. EC Dysfunction Assay

Cell culture medium was collected to measure the secreted levels of ET-1, NO, and PGI_2_ using an ET-1 ELISA kit (Shanghai Enzyme Biotechnology Co., Ltd., Shanghai, China), following the manufacturer's protocol.

To determine the intracellular NO level, we incubated EA.hy926 cells with the NO-specific fluorescent dye DAF-FM DA (50 *μ*M, Beyotime Institute of Biotechnology) in culture medium without phenol red at 37°C for 30 min after treatment with LSS, LSS+DHWE (0.1, 1, and 10 mg/L), or not. The EA.hy926 cells were washed in PBS twice after the fluorescent dye treatment and then photographed and analysed via fluorescence microscopy. Images were captured under fluorescence microscopy, and the fluorescence intensity was quantified from at least three random fields per slide from three slides.

### 2.16. Oxidative Stress Assay

EA.hy926 cells were collected to measure secreted SOD activity, MDA content, GSH content, and GSSG content using the ELISA kit (Beijing Solarbio Science & Technology Co., Ltd., Beijing, China), following the manufacturer's instructions.

To determine the intracellular ROS level, we incubated EA.hy926 cells with the ROS-specific fluorescent dye DHE (50 *μ*M, Beyotime Institute of Biotechnology, China) in culture medium without phenol red at 37°C for 30 min after treatment with LSS, LSS+DHWE (0.1, 1, and 10 mg/L), or not. The EA.hy926 cells were washed in PBS twice after the fluorescent dye treatment and then photographed and analysed via fluorescence microscopy. Images were captured under fluorescence microscopy, and the fluorescence intensity was quantified from at least three random fields per slide from three slides.

### 2.17. Quantitative Real-Time PCR

Total RNA of the cells was extracted using the TRIzol reagent (Invitrogen, Carlsbad, CA, USA), according to the manufacturer's instructions. The RNA was reverse transcribed using the PrimeScript RT Master Mix (Perfect Real Time), following the manufacturer's protocol. The resultant cDNA was applied as the template for quantitative PCR analyses in the Thermal Cycler Dice® Real Time System (Takara Bio Inc., Shiga, Japan) with the following sets of primers: primers for quantitative real-time PCR (qPCR) were designed by the Primer3 software and are listed in Table 1. The mRNA expression data are expressed as relative expression ratio normalized to GAPDH.

### 2.18. Statistical Analysis

Data are presented as the mean ± standard deviation. Statistical differences were determined using analysis of variance (ANOVA), where *p* < 0.05 was considered statistically significant. The analyses were performed using the Statistical Program for Social Sciences Software (IBM SPSS, International Business Machines Corporation, Armonk City, NY, United States).

## 3. Results

### 3.1. DHWE Reduce Plaque Formation in Zebrafish Larval AS Model

**Figure 1:**
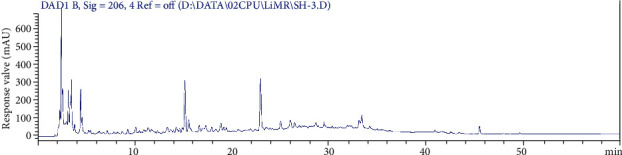
HPLC profile of DHWE.

**Figure 2:**
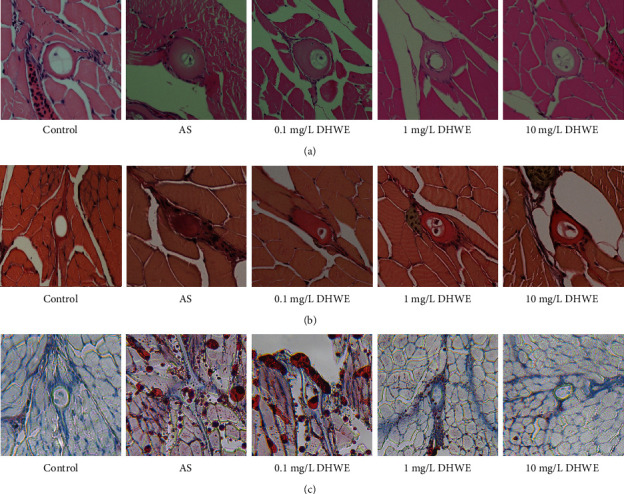
DHWE reduced the AS in AS zebrafish. (a) HE staining results showed that DHWE reduce vascular stenosis in AS zebrafish. (b) EVG staining results showed that DHWE reduced the fibrosis of plaques in AS zebrafish blood vessels. (c) Oil red O staining results showed that DHWE reduced the lipids in the plaques in the blood vessels of AS zebrafish.

DHWE were analysed with HPLC (Figure 1). The plaques in the blood vessels of each group of zebrafish were detected by HE staining, EVG staining, and oil red O staining. There were no plaques were observed in the blood vessels of zebrafish in the control group (Figure 2). Compared to the control group, a large number of plaques appeared in the blood vessels of the zebrafish in the AS group, resulting in vascular stenosis and a large amount of lipids in the plaques. However, 1 mg/L and 10 mg/L DHWE significantly improved the plaque formation in AS zebrafish blood vessels, but 0.1 mg/L DHWE showed no obvious effect.

### 3.2. DHWE Reduced the Early AS Symptoms in Zebrafish Larval AS Model

**Figure 3:**
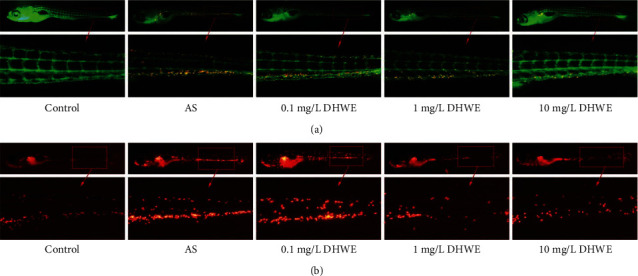
DHWE reduced the early AS in AS zebrafish. (a) DHWE reduced cholesterol accumulation in AS zebrafish blood vessels. (b) DHWE reduced macrophage infiltration in AS zebrafish blood vessels.

The main symptoms of early AS are macrophage infiltration and cholesterol accumulation in blood vessels. Therefore, macrophage infiltration and cholesterol accumulation in zebrafish blood vessels were detected to show the anti-AS efficacy of DHEW. Results showed that macrophage infiltration and cholesterol accumulation rarely occurred in the blood vessels of zebrafish in the control group; in contrast, macrophage infiltration and cholesterol accumulation were abundant in the blood vessels of the zebrafish in the AS group (Figure 3). Compared with the AS group, 1 mg/L and 10 mg/L DHWE significantly reduced macrophage infiltration and cholesterol accumulation in AS zebrafish blood vessels, but 0.1 mg/L DHWE did not. Therefore, these results indicate that DHWE can effectively prevent early AS.

### 3.3. DHWE Reduced Lipid Levels in AS Zebrafish

**Figure 4:**
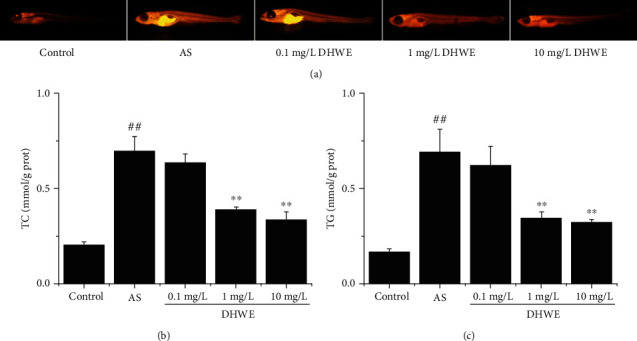
DHWE reduced lipid accumulation in AS zebrafish. (a) The results of Nile red staining showed that DHWE reduced the blood lipid level in AS zebrafish. DHWE reduced the (b) TC content and (c) TG content in AS zebrafish. ^#^*p* < 0.05 and ^##^*p* < 0.01 compared to the control group and ^∗^*p* < 0.05 and ^∗∗^*p* < 0.01 compared to the AS group.

Hyperlipidemia is one of the main causes of AS, so this study detects the lipid level of zebrafish to show the lipid-lowering effect of DHWE. Lipid levels were detected by Nile red staining. Compared with the control group, the lipid level significantly increased in the AS group, which was notably reversed by 1 mg/L and 10 mg/L DHWE (Figure 4(a)). The same results were also found for the levels of TG and TC in AS zebrafish (Figures 4(b) and 4(c)). The levels of TG and TC significantly increased in the AS group. Treatment with 1 mg/L and 10 mg/L DHWE significantly decreased the levels of TG and TC, while 0.1 mg/L DHWE did not effectively decrease the levels of TG and TC. In summary, DHWE could efficiently reduce lipid levels in AS zebrafish.

### 3.4. DHWE Improved Oxidative Stress in AS Zebrafish

**Figure 5:**
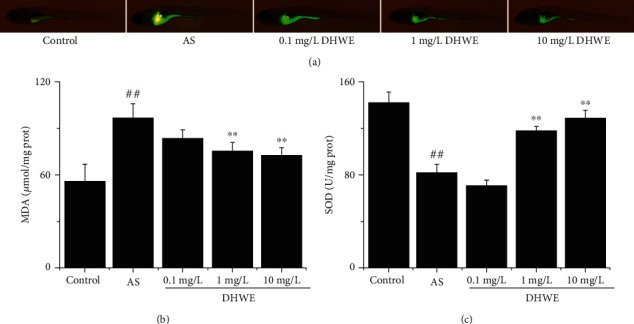
DHWE reduced oxidative stress in AS zebrafish. (a) The results of DCFH-DA assay showed that DHWE reduced the ROS activity in AS zebrafish. (b) DHWE reduced MDA content in AS zebrafish. (c) DHWE increased SOD activity in AS zebrafish. ^#^*p* < 0.05 and ^##^*p* < 0.01 compared to the control group and ^∗^*p* < 0.05 and ^∗∗^*p* < 0.01 compared to the AS group.

As shown in Figure 5(a), ROS levels significantly increased in the AS group in comparison with the control group. Treatment with 1 mg/L and 10 mg/L DHWE markedly decreased ROS levels, while 0.1 mg/L DHWE did not reverse the ROS increase. With SOD activity and MDA content, the same reverse was induced by DHWE in zebrafish. As shown in Figures 5(b) and 5(c), treatment with 1 mg/L and 10 mg/L DHWE significantly reduced MDA content and significantly increased SOD activity, while 0.1 mg/L DHWE had no significant effect on SOD activity and MDA content.

### 3.5. DHWE Reduced Inflammation in AS Zebrafish

**Figure 6:**
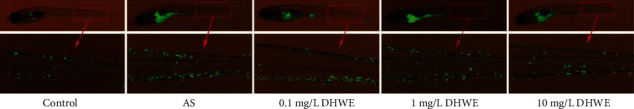
DHWE reduced the number of neutrophils in AS zebrafish.

The number of neutrophils can reflect the degree of inflammation in AS zebrafish. As shown in Figure 6, different with only a very small amount of neutrophils in the control group, a much larger number of neutrophils appeared in the blood vessels of AS zebrafish. After treatment with 1 mg/L and 10 mg/L DHWE, the number of neutrophils significantly decreased, which was not observed in the 0.1 mg/L DHWE group. These results indicate that DHWE can effectively reduce inflammation in AS zebrafish.

### 3.6. DHWE Improved LSS-Induced EC Dysfunction

**Figure 7:**
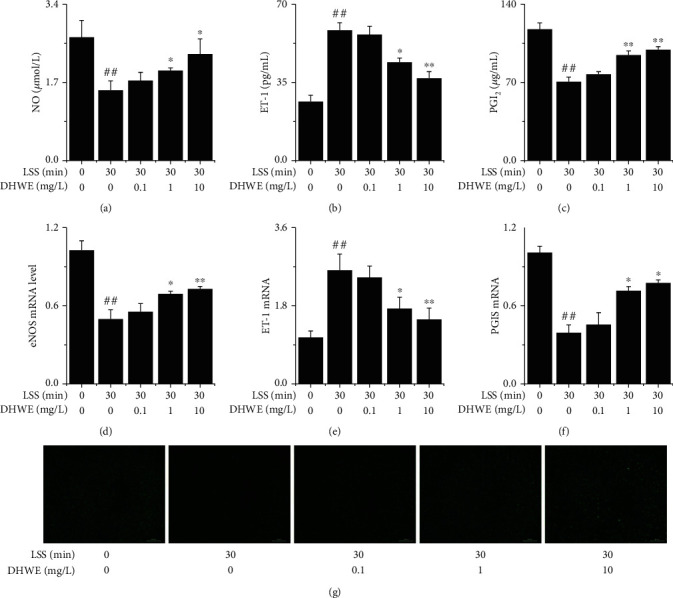
DHWE improved LSS-induced EC dysfunction. (a) DHWE increased the LSS-induced decrease in NO release from EA.hy926 cells. (b) DHWE decreased the LSS-induced increase in ET-1 release from EA.hy926 cells. (c) DHWE increased the LSS-induced decrease in PGI_2_ release from EA.hy926 cells. (d) DHWE increased the LSS-induced decrease of the eNOS mRNA level. (e) DHWE decreased the LSS-induced increase of the ET-1 mRNA level. (f) DHWE increased the LSS-induced decrease of the PGIS mRNA level. (g) The results of the DAF-FM DA assay showed that DHWE increase the LSS-induced decrease in NO activity in EA.hy926 cells. ^##^*p* < 0.01 compared to 0 min LSS + 0 *μ*g/mL DHWE; ^∗^*p* < 0.05 and ^∗∗^*p* < 0.01 compared to 30 min LSS + 0 *μ*g/mL DHWE.

Referring to our previous research [26], EA.hy926 cells were exposed to laminar flow with a value of 0 or 3 dyn/cm^2^ for 30 min. LSS significantly reduced the release of NO and PGI_2_ and significantly increased the release of ET-1, which was significantly inhibited by 1 mg/L and 10 mg/L DHWE but not by 0.1 mg/L DHWE (Figures 7(a) – 7(c)). We also detected changes in the mRNA expression of key genes, such as *ET-1*, *eNOS*, and *PGIS*, by qRT-PCR in EA.hy926 cells. LSS significantly reduced the mRNA levels of *eNOS* and *PGIS* and significantly increased the *ET-1* mRNA level, which was significantly reversed by 1 mg/L and 10 mg/L DHWE but not by 0.1 mg/L DHWE (Figures 7(d) – 7(f)). Then, we examined intracellular NO activities using the fluorescent probe DAF-FM DA. As shown in Figure 7(g), LSS significantly reduced NO activity in EA.hy926 cells, and 1 mg/L and 10 mg/L DHWE significantly increased NO activity, but 0.1 mg/L DHWE did not improve the LSS-induced decrease in NO activity. To summarize, DHWE led to an effective improvement in LSS-induced EC dysfunction.

### 3.7. DHWE Improved LSS-Induced Oxidative Stress

**Figure 8:**
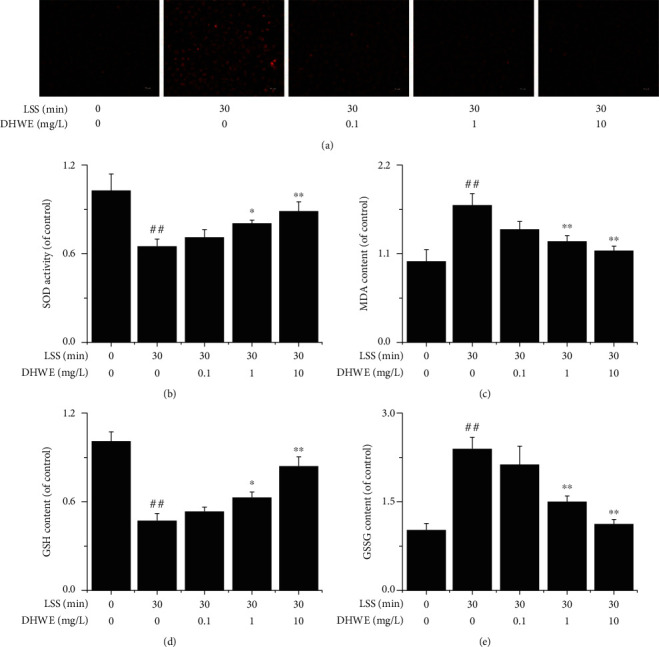
DHWE alleviated LSS-induced oxidative stress. (a) The results of the DCFH-DA assay showed that DHWE attenuate the LSS-induced increase in ROS activity in EA.hy926 cells. (b) DHWE increased the LSS-induced decrease in SOD activity in EA.hy926 cells. (c) DHWE decreased the LSS-induced increase in MDA content in EA.hy926 cells. (d) DHWE increased the LSS-induced decrease in GSH content in EA.hy926 cells. (e) DHWE decreased the LSS-induced increase in GSSG content in EA.hy926 cells. ^##^*p* < 0.01 compared to 0 min LSS + 0 *μ*g/mL DHWE; ^∗^*p* < 0.05 and ^∗∗^*p* < 0.01 compared to 30 min LSS + 0 *μ*g/mL DHWE.

We examined intracellular ROS activity using the fluorescent probe DHE. LSS significantly induced ROS activity, which was significantly inhibited by 1 mg/L and 10 mg/L DHWE but not by 0.1 mg/L DHWE (Figure 8(a)). We also assessed the levels of SOD, MDA, GSSG, and GSH in EA.hy926 cells. As shown in Figures 8(b) – 8(e), LSS significantly reduced SOD and GSH levels and significantly increased MDA and GSSG levels, which was significantly reversed by 1 mg/L and 10 mg/L DHWE but not by 0.1 mg/L DHWE. According to reference [27], we calculated the redox ratios of each group. As shown in Table 2, LSS significantly reduced the redox ratio, while DHWE treatment significantly increased the redox ratio. To summarize, DHWE led to an effective improvement in LSS-induced oxidative stress.

### 3.8. DHWE Improved LSS-Induced Inflammation

**Figure 9:**
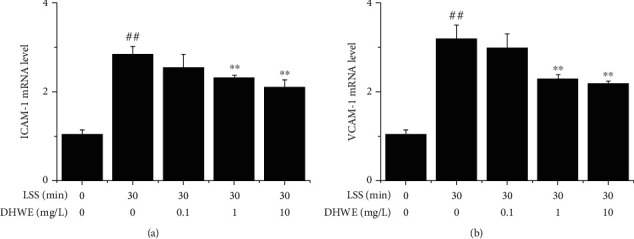
DHWE improved LSS-induced inflammation. DHWE decreased the LSS-induced increase in (a) ICAM-1 and (b) VCAM-1 mRNA levels. ^##^*p* < 0.01 compared to 0 min LSS + 0 *μ*g/mL DHWE; ^∗∗^*p* < 0.01 compared to 30 min LSS + 0 *μ*g/mL DHWE.

We also detected changes in the mRNA expression of key molecules, such as *ICAM-1* and *VCAM-1*, by qRT-PCR in EA.hy926 cells in response to inflammation. As shown in Figure 9, 1 mg/L and 10 mg/L DHWE significantly repressed the LSS-induced increase in mRNA levels of *ICAM-1* and *VCAM-1*. To sum it up, DHWE led to an effective improvement in LSS-induced inflammation.

## 4. Discussion

Zebrafish are a unique vertebrate model that has characteristics of invertebrate models (small size, powerful genetic tractability, high fecundity, ease of maintenance, and relatively low cost) and the advantage of evolutionary conservation in mammals. Thus, zebrafish are invaluable for studying vertebrate development and physiology as well as for modelling human diseases, such as convulsion, epilepsy, and nonalcoholic fatty liver disease [28–30].

Zebrafish fed with high-cholesterol diet (HCD) is the most commonly used zebrafish early AS model [31]. In the present study, we found a large amount of cholesterol accumulation in the blood vessels of the HCD zebrafish model, which is in accord with studies about lipid accumulation in blood vessels and early plaques of AS [23]. We also observed a large number of macrophage infiltration in the blood vessels of AS zebrafish, which is similar to the early symptoms of human atherosclerosis. These characteristics indicate that AS zebrafish model would be a suitable model for early AS mechanism research and drug development. In this study, DHWE significantly improved lipid accumulation and macrophage infiltration in the blood vessels of AS zebrafish. These results indicate that DHWE can reduce the early AS. Plaque formation in blood vessels is a hallmark of AS. In this study, we found that after feeding zebrafish for 45 days of HCD, plaques appeared in the blood vessels of zebrafish, which caused vascular stenosis. And the results of EVG staining and oil red O staining show that these plaques are mainly composed of fibrous tissue and lipids, which are similar to plaques in AS patients. Interestingly, DHWE significantly improved the plaque formation in AS zebrafish blood vessels. These results indicate that DHWE can relieve the progression of AS.

Hypercholesterolemia is an important risk factor for atherosclerosis, so HCD is also a common method for constructing AS animal model [32]. Excess cholesterol in the blood will accumulate on the walls of blood vessels and eventually form plaques. Excessive cholesterol can also cause liver damage, induce liver lipid metabolism dysfunction, further increase the accumulation of cholesterol, and promote the occurrence of AS [22]. In the present study, we found that HCD induces a large amount of cholesterol to accumulate in the blood vessels of zebrafish, while also increasing the content of lipids, TC and TG, causing lipid metabolism disorders. Interestingly, DHWE significantly reduced cholesterol accumulation and lipid metabolism dysfunction induced by HCD.

In addition to hypercholesterolemia, inflammation and oxidative stress are also involved in the occurrence of AS [33]. In the early stage of AS, a large number of neutrophils migrate to the vascular endothelium, causing vascular inflammation, and at the same time release a large amount of ROS, causing vascular damage, and ultimately promote the occurrence of AS [22]. In the present study, we found that a large number of neutrophils were recruited in the blood vessels of AS zebrafish. And AS zebrafish also showed symptoms of oxidative stress, mainly manifested as follows: ROS activity and MDA content increased significantly, while SOD activity decreased significantly. Interestingly, DHWE significantly improved the HCD-induced oxidative stress and inflammation. These results also indicate that the anti-AS effect of DHWE may be related to its antioxidant and anti-inflammatory effects.

Some studies have found that LSS plays an important role in the occurrence of AS [34]. LSS destroys the balance of the release of some vasoactive substances in vascular endothelial cells, causing EC dysfunction, which is the main cause of AS [35]. EC dysfunction is characterized by an imbalance between vasodilatory and vasoconstrictive molecules. These molecules are synthesized and released by ECs and include PGI_2_, NO, and ET-1. PGI_2_ and NO are effective vasodilators and are produced by PGIS and eNOS, respectively, while ET-1 is a potent vasoconstrictor [36]. Additionally, high levels of ET-1 are considered one of the risk factors for AS [37]. In the present study, we used a parallel flow chamber to establish an LSS-induced EC dysfunction model. We found that LSS significantly reduced the release of NO and PGI_2_ and significantly increased the release of ET-1. We also found that LSS significantly reduced the mRNA levels of *eNOS* and *PGIS* and significantly increased the *ET-1* mRNA level. Intriguingly, DHWE significantly improved the EC dysfunction phenomena induced by LSS. These results indicate that DHWE may inhibit the occurrence of AS by improving LSS-induced EC dysfunction.

LSS can also cause oxidative stress and promote the occurrence of AS [26]. In the present study, we found that LSS significantly reduced the activity of SOD and GSH and significantly increased the levels of ROS, MDA, and GSSG, breaking the “oxidation-reduction” balance of ECs. Intriguingly, DHWE significantly improved the oxidative stress phenomena induced by LSS. These results indicate that DHWE may inhibit the occurrence of AS by improving LSS-induced oxidative stress.

Atherosclerosis is a chronic inflammatory disease in which a variety of immune cells are involved, and neutrophils are closely related to the initiation of early chronic inflammation in AS. Abnormal expression of adhesion molecules (e.g., ICAM-1 and VCAM-1) in endothelial cells causes neutrophil recruitment and mediates the migration of neutrophils to vascular inflammation sites, accelerating the development of AS vascular wall inflammation [38]. In the present study, we found that LSS significantly increased the mRNA expression of *ICAM-1* and *VCAM-1*. Furthermore, a large number of neutrophils were recruited to the blood vessels of AS zebrafish exposed to LSS. These results suggest that LSS may mediate neutrophil recruitment by increasing the expression of ICAM-1 and VCAM-1, ultimately accelerating the development of AS vascular wall inflammation. Intriguingly, DHWE significantly improved the inflammation phenomena induced by LSS. These results indicate that DHWE may inhibit the occurrence of AS by improving LSS-induced inflammation.

In our previous studies [22], we have observed that aspirin and vitamin C can reduce HCD-induced oxidative stress and inflammation, but cannot improve HCD-induced lipid metabolism disorders, and ultimately, the effect of inhibiting plaque formation is minimal. Unlike these clinical drugs, DHWE have a better anti-AS effect. DHWE significantly reduce HCD-induced oxidative stress, inflammation, and lipid metabolism disorders and ultimately effectively reduce the formation of plaque.

## 5. Conclusions

In summary, DHWE alleviate HCD-induced cholesterol accumulation, lipid metabolism disorder, and oxidative stress, while also reducing the EC dysfunction induced by LSS, and ultimately reduces the formation of plaque in blood vessels. The results of this study indicate that DH may be used as a drug to prevent AS. However, this study still has some limitations. For example, this study is only a preliminary study of the anti-AS efficacy of DHWE, but not its molecular mechanism of action. Moreover, this study only studied the crude extract of DH and did not clarify the specific active substances. In future research, we will study the specific active ingredients of DH anti-AS and study its mechanism of action.

## Figures and Tables

**Table 1:** Primer sequence.

Gene	Species		Primer sequence (5′→3′)
ICAM-1	Homo	Forward	TCTTCCTCGGCCTTCCCATA
		Reverse	AGGTACCATGGCCCCAAATG

VCAM-1	Homo	Forward	GGACCACATCTACGCTGACA
		Reverse	TTGACTGTGATCGGCTTCCC

ET-1	Homo	Forward	GGCTGAAGGATCGCTTTGAGA
		Reverse	GCTCAGCGCCTAAGACTGTTT

eNOS	Homo	Forward	CTGGCTACAAGCACCGTGA
		Reverse	GGTTTCCAGCCCTGCTGTAT

PGIS	Homo	Forward	ATTACAACATGCCCTGGGGG
		Reverse	TGCGTTGATCAGCTCCAAGT

GAPDH	Homo	Forward	CCATGGGGAAGGTGAAGGTC
		Reverse	GCGCCCAATACGACCAAATC

**Table 2:** The redox ratios of each group.

Group	[GSH]^2^/[GSSG]
Control	1.036351178 ± 0.27462764
AS	0.092359845 ± 0.017842737^##^
0.1 mg/L DHWE	0.131603581 ± 0.005921076^∗^
1 mg/L DHWE	0.256627824 ± 0.042547553^∗∗^
10 mg/L DHWE	0.627796922 ± 0.058491923^∗∗^

Note: ^##^*p* < 0.01 compared to the control group and ^∗^*p* < 0.05 and ^∗∗^*p* < 0.01 compared to the AS group.

## Data Availability

All data used to support the findings of this study are included within the article.

## Abbreviations

DH: Dendrobium catenatum Lindl.
DHWE: Dendrobium catenatum Lindl. water extracts
AS: Atherosclerosis
HCD: High-cholesterol diet
EC: Endothelial cell
LSS: Low shear stress
ROS: Reactive oxygen species
SOD: Superoxide dismutase
MDA: Malondialdehyde
TG: Triglyceride
TC: Total cholesterol
GSH: Glutathione
GSSG: Glutathiol
NO: Nitric oxide
ET-1: Endothelin-1
PGI_2_: Epoprostenol
eNOS: Endothelial nitric oxide synthase
ICAM-1: Intercellular adhesion molecule-1
VCAM-1: Vascular cell adhesion molecule-1.

## Supplementary Materials

Supplementary Materials (See the Supplementary Materials 1 for specific steps), EVG staining. (See the Supplementary Materials 2 for specific steps) and oil red O staining. (See the Supplementary Materials 3 for specific steps).

## References

[1] Hu Y., Davison F., Zhang Z., Xu Q. (2003). Endothelial replacement and angiogenesis in arteriosclerotic lesions of allografts are contributed by circulating progenitor cells. *Circulation.*.

[2] Foteinos G., Afzal A. R., Mandal K., Jahangiri M., Xu Q. (2005). Anti-heat shock protein 60 autoantibodies induce atherosclerosis in apolipoprotein E-deficient mice via endothelial damage. *Circulation.*.

[3] Ma X., Feng Y. (2016). Hypercholesterolemia tunes hematopoietic stem/progenitor cells for inflammation and atherosclerosis. *International Journal of Molecular Sciences*.

[4] Karbiner M. S., Sierra L., Minahk C., Fonio M. C., Bruno M. P., Jerez S. (2013). The role of oxidative stress in alterations of hematological parameters and inflammatory markers induced by early hypercholesterolemia. *Life Sciences*.

[5] Libby P., Buring J. E., Badimon L. (2019). Atherosclerosis. *Nature Reviews Disease Primers*.

[6] Guo F. X., Hu Y. W., Zheng L., Wang Q. (2017). Shear stress in autophagy and its possible mechanisms in the process of atherosclerosis. *DNA and Cell Biology*.

[7] Lehoux S., Jones E. A. (2016). Shear stress, arterial identity and atherosclerosis. *Thrombosis and Haemostasis*.

[8] Gongol B., Marin T., Zhang J. (2019). Shear stress regulation of miR-93 and miR-484 maturation through nucleolin. *Proceedings of the National Academy of Sciences*.

[9] Wang L., Luo J. Y., Li B. (2016). Integrin-YAP/TAZ-JNK cascade mediates atheroprotective effect of unidirectional shear flow. *Nature.*.

[10] Mahmoud M. M., Kim H. R., Xing R. (2016). TWIST1 integrates endothelial responses to flow in vascular dysfunction and atherosclerosis. *Circulation Research*.

[11] Writing Group Members, Mozaffarian D., Benjamin E. J. (2016). Heart disease and stroke statistics-2016 update: a report from the American Heart Association. *Circulation.*.

[12] Lozano R., Naghavi M., Foreman K. (2012). Global and regional mortality from 235 causes of death for 20 age groups in 1990 and 2010: a systematic analysis for the Global Burden of Disease Study 2010. *Lancet.*.

[13] Wierzbicki A. S., Hardman T. C., Viljoen A. (2012). New lipid-lowering drugs: an update. *International Journal of Clinical Practice*.

[14] Nording H., Baron L., Langer H. F. (2020). Platelets as therapeutic targets to prevent atherosclerosis. *Atherosclerosis.*.

[15] Zhang S., Li L., Chen W., Xu S., Feng X., Zhang L. (2021). Natural products: the role and mechanism in low-density lipoprotein oxidation and atherosclerosis. *Phytotherapy Research*.

[16] Ozkanlar S., Akcay F. (2012). Antioxidant vitamins in atherosclerosis--animal experiments and clinical studies. *Advances in Clinical and Experimental Medicine*.

[17] Chan K. C., Ho H. H., Lin M. C. (2014). Mulberry water extracts inhibit rabbit atherosclerosis through stimulation of vascular smooth muscle cell apoptosis via activating p 53 and regulating both intrinsic and extrinsic pathways. *Journal of Agricultural and Food Chemistry*.

[18] Ko M., Oh G. T., Park J., Kwon H. J. (2020). Extract of high hydrostatic pressure-treated danshen (Salvia miltiorrhiza) ameliorates atherosclerosis via autophagy induction. *BMB Rep.*.

[19] Su S. Q., Jiang H., Li Q. M., Zha X. Q., Luo J. P. (2020). Study on chemical constituents of Dendrobium huoshanense stems and their anti-inflammatory activity. *Zhongguo Zhong Yao Za Zhi.*.

[20] Li Q. M., Jiang H., Zha X. Q. (2020). Anti-inflammatory bibenzyls from the stems ofDendrobium huoshanensevia bioassay guided isolation. *Natural Product Research*.

[21] Wang Y. H. (2021). Traditional uses, chemical constituents, pharmacological activities, and toxicological effects of _Dendrobium_ leaves: A review. *Journal of Ethnopharmacology*.

[22] Han J. (2021). Zebrafish Model for Screening Antiatherosclerosis Drugs. *Oxidative Medicine and Cellular Longevity*.

[23] Fang L., Green S. R., Baek J. S. (2011). In vivo visualization and attenuation of oxidized lipid accumulation in hypercholesterolemic zebrafish. *Journal of Clinical Investigation*.

[24] Yan B., Han P., Pan L. (2014). IL-1*β* and reactive oxygen species differentially regulate neutrophil directional migration and Basal random motility in a zebrafish injury-induced inflammation model. *The Journal of Immunology*.

[25] Yang J., Kim G., Chae J. (2019). Antioxidant and anti-inflammatory effects of an ethanol fraction from the Schisandra chinensis baillon hot water extract fermented using Lactobacilius paracasei subsp. tolerans. *Food Science and Biotechnology*.

[26] Fan X., Han J., Zhu L. (2020). Protective Activities of Dendrobium huoshanense C. Z. Tang et S. J. Cheng Polysaccharide against High-Cholesterol Diet-Induced Atherosclerosis in Zebrafish. *Oxidative Medicine and Cellular Longevity*.

[27] Zalewska A., Szarmach I., Żendzian-Piotrowska M., Maciejczyk M. (2020). The effect of N-acetylcysteine on respiratory enzymes, ADP/ATP ratio, glutathione metabolism, and nitrosative stress in the salivary gland mitochondria of insulin resistant rats. *Nutrients.*.

[28] Sharma P., Kumari S., Sharma J., Purohit R., Singh D. (2021). Hesperidin interacts with CREB-BDNF signaling pathway to suppress pentylenetetrazole-induced convulsions in zebrafish. *Frontiers in Pharmacology*.

[29] Tanwar G., Mazumder A. G., Bhardwaj V. (2019). Target identification, screening and _in vivo_ evaluation of pyrrolone-fused benzosuberene compounds against human epilepsy using Zebrafish model of pentylenetetrazol-induced seizures. *Scientific Reports*.

[30] Ma J., Yin H., Li M. (2019). A comprehensive study of high cholesterol diet-induced larval zebrafish model: a Short-TimeIn VivoScreening method for non-alcoholic fatty liver disease drugs. *International Journal of Biological Sciences*.

[31] Stoletov K., Fang L., Choi S. H. (2009). Vascular lipid accumulation, lipoprotein oxidation, and macrophage lipid uptake in hypercholesterolemic zebrafish. *Circulation Research*.

[32] Lu H., Daugherty A. (2015). Atherosclerosis. *Arteriosclerosis, Thrombosis, and Vascular Biology*.

[33] Yuan T., Yang T., Chen H. (2019). New insights into oxidative stress and inflammation during diabetes mellitus- accelerated atherosclerosis. *Redox Biology*.

[34] Heo K. S., Fujiwara K., Abe J. (2014). Shear stress and atherosclerosis. *Molecules and Cells*.

[35] Baeyens N. (2018). Fluid shear stress sensing in vascular homeostasis and remodeling: towards the development of innovative pharmacological approaches to treat vascular dysfunction. *Biochemical Pharmacology*.

[36] Sharma P., Dong Y., Somers V. K. (2018). Intermittent hypoxia regulates vasoactive molecules and alters insulin- signaling in vascular endothelial cells. *Scientific Reports*.

[37] Zeiher A. M., Goebel H., Schächinger V., Ihling C. (1995). Tissue endothelin-1 immunoreactivity in the active coronary atherosclerotic plaque. A clue to the mechanism of increased vasoreactivity of the culprit lesion in unstable angina. *Circulation.*.

[38] Döring Y., Soehnlein O., Weber C. (2017). Neutrophil extracellular traps in atherosclerosis and atherothrombosis. *Circulation Research*.

