# Supplementary material for: Dendrobium catenatum Lindl. Water Extracts Attenuate Atherosclerosis
Source: Mediators Inflamm. 2021 Aug 24;2021:9951946. doi: 10.1155/2021/9951946 (PMC8407999; doi:10.1155/2021/9951946)
Supplement: Supplementary Materials — (See the Supplementary Materials 1 for specific steps), EVG staining. (See the Supplementary Materials 2 for specific steps) and oil red O staining. (See the Supplementary Materials 3 for specific steps). [file 9951946.f1.zip › Supplementary Materials 1 (1).docx]

**H&E staining**

| **Reagent** | **Manufacturer** | **Cat.log** |
| --- | --- | --- |
| Xylene  Alcohol  H&E staining kit  Differentiating solution  Ammonia solution  Resin | Sinopharm  Sinopharm  Ribiology  Ribiology  Ribiology  Sinopharm | 10023418  100092683  10004160 |

1. Deparaffinize and hydrate to water: Process slides according to below.

Xylene I--20 min

Xylene II--20min

100% alcohol I--5 min

100% alcohol II--5 min

75% alcohol--5 min

Rinse in water

1. Stain in hematoxylin solution: Immerse slides in hematoxylin solution for 3 to 5 min, rinse them in water. Then differentiate sections with acid alcohol, rinse again. Blue up sections with ammonia solution, wash in slowly running tap water.
2. Stain in eosin:

85% alcohol I--5 min

95% alcohol II--5 min

Eosin--5min

1. Dehydrate and mount:

100% alcohol I--5 min

100% alcohol II--5 min

100% alcohol III--5 min

Xylene I--5 min

Xylene II--5 min

Mount withresin

Results:

Nucleus-----------blue

Cytoplasm--------red
