# Supplementary material for: Dendrobium catenatum Lindl. Water Extracts Attenuate Atherosclerosis
Source: Mediators Inflamm. 2021 Aug 24;2021:9951946. doi: 10.1155/2021/9951946 (PMC8407999; doi:10.1155/2021/9951946)
Supplement: Supplementary Materials — (See the Supplementary Materials 1 for specific steps), EVG staining. (See the Supplementary Materials 2 for specific steps) and oil red O staining. (See the Supplementary Materials 3 for specific steps). [file 9951946.f1.zip › Supplementary Materials 2 (1).docx]

**EVG staining**

| **Reagent** | **Manufacturer** | **Cat.log** |
| --- | --- | --- |
| Xylene  Alcohol  EVG staining kit  Resin | Sinopharm  Sinopharm  Ribiology  Sinopharm | 10023418  100092683  10004160 |

1. Deparaffinize and hydrate to water: Process slides according to below.

Xylene I--20 min

Xylene II--20min

100% alcohol I--5 min

100% alcohol II--5 min

75% alcohol--5 min

Rinse in water

1. EVG stain: Immerse slides in EVG staining solution for 30 min, wash in tap water.
2. Differentiation: Differentiate in FeCl_3_ solution slightly, and then wash in tap water. Repeat differentiation if needed (check slides with microscope to make sure appropriate differentiation, the background should be pale or white).
3. VG stain: Immerse slides in VG solution for 1 to 3 min, wash in running tap water quickly.
4. Dehydrate and mount:

100% alcohol I--5 min

100% alcohol II--5 min

100% alcohol III--5 min

Xylene I--5 min

Mount withresin

Results:

Elastic fibers---------------------------------dark purple

Collagen fibers------------------------------red
