# Supplementary material for: Dendrobium catenatum Lindl. Water Extracts Attenuate Atherosclerosis
Source: Mediators Inflamm. 2021 Aug 24;2021:9951946. doi: 10.1155/2021/9951946 (PMC8407999; doi:10.1155/2021/9951946)
Supplement: Supplementary Materials — (See the Supplementary Materials 1 for specific steps), EVG staining. (See the Supplementary Materials 2 for specific steps) and oil red O staining. (See the Supplementary Materials 3 for specific steps). [file 9951946.f1.zip › Supplementary Materials 3 (1).docx]

**Oil Red O stainingfor slides**

| **Reagent** | **Manufacturer** | **Cat.log** |
| --- | --- | --- |
| Fixative  ORO staining kit  Alcohol  Hematoxylin solution  Differentiating solution  Ammonia solution  Glycerogelatin | Ribiology  Ribiology  Sinopharm  Ribiology  Ribiology  Ribiology  Ribiology | 100092683 |

1. Dry frozen sections in room temperature, place in fixative for 15min. Wash in running tap water. Air-dry.
2. Place sections in ORO solution for 8-10min (in dark room), wash in distill water.
3. Differentiate slightly with 75% alcohol.
4. Stain in hematoxylin solution: Immerse slides in hematoxylin solution for 3 to 5 min, rinse them in water. Then differentiate sections with acid alcohol, rinse again. Blue up sections with ammonia solution, wash in slowly running tap water.
5. Mount with glycerogelatin.

Results:

Lipids-----------------red

Nucleus--------------blue
